# Ruptured Heterotopic Pregnancy: A Rare Encounter in Acute Surgical Care Settings

**DOI:** 10.7759/cureus.11782

**Published:** 2020-11-30

**Authors:** Amro Elhadidi, Abdelrazak Alhariri, Mohamed Hosny Garib, Ahmed Mansour, Bandar Almutiri

**Affiliations:** 1 Surgery, Mansoura Univeristy, Mansoura, EGY; 2 Surgery, Buriadah Central Hospital, Buriadah, SAU

**Keywords:** heterotopic pregnancy, ectopic pregnancy, salpingectomy, hemorrhagic shock

## Abstract

Heterotopic pregnancy is a rare, life-threatening clinical entity with an overall incidence of about 1:30,000 in spontaneous natural conception cases, especially in cases of delayed diagnosis or conflicting clinical features. Here, we present an unusual case of heterotopic pregnancy in a 22-year-old multigravida presented to the emergency department (ED) with a clinical picture of the acute abdomen following recent abdominal trauma. Abdominal ultrasound revealed hemoperitoneum and a single viable intrauterine pregnancy at seven weeks' gestation. Following surgical exploration, the patient underwent removal of the ectopic pregnancy tissue with right salpingectomy. Since the presence of a conflicting history or equivocal physical signs and symptoms makes it difficult to diagnose heterotopic pregnancy, ED physicians and surgeons must consider the diagnosis even when dealing with viable intrauterine pregnancies. Besides, acute abdominal pain associated with shock should be regarded as suggestive of heterotopic pregnancy. Thus, prompt evaluation and a high index of suspicion are of paramount importance to prevent unwanted sequelae.

## Introduction

Heterotopic pregnancy is a rare gynecological entity characterized by simultaneous intrauterine and extrauterine pregnancies [1]. The incidence of heterotopic pregnancy, which is estimated at about 1:7,000 to 1:50,000 pregnancies in the general population, has been reported to be 0.09% to 1.00% of pregnancies when using assisted fertilization techniques [2-5]. 

The risk factors associated with heterotopic pregnancy may include pelvic inflammatory disease, previous history of ectopic pregnancies, personalized assisted fertilization, and ovarian hyperstimulation with increase in the risk of abortion with an estimated odd ratio of 3.948 [6,7].

Heterotopic pregnancy has a wide variety of clinical presentations, ranging from slight abdominal pain with nausea and vomiting to hemorrhagic shock with or without evidence of adnexal mass rupture [2,7]. Hence, ED physicians and acute care surgeons should maintain a high index of suspicion for this fatal condition to allow for accurate early diagnosis and proper management [8].

Herein, we present a case of heterotopic pregnancy in a patient who had a vague history of recent abdominal trauma without any risk factors, whose initial diagnosis was difficult to make

## Case presentation

**Figure 1:**
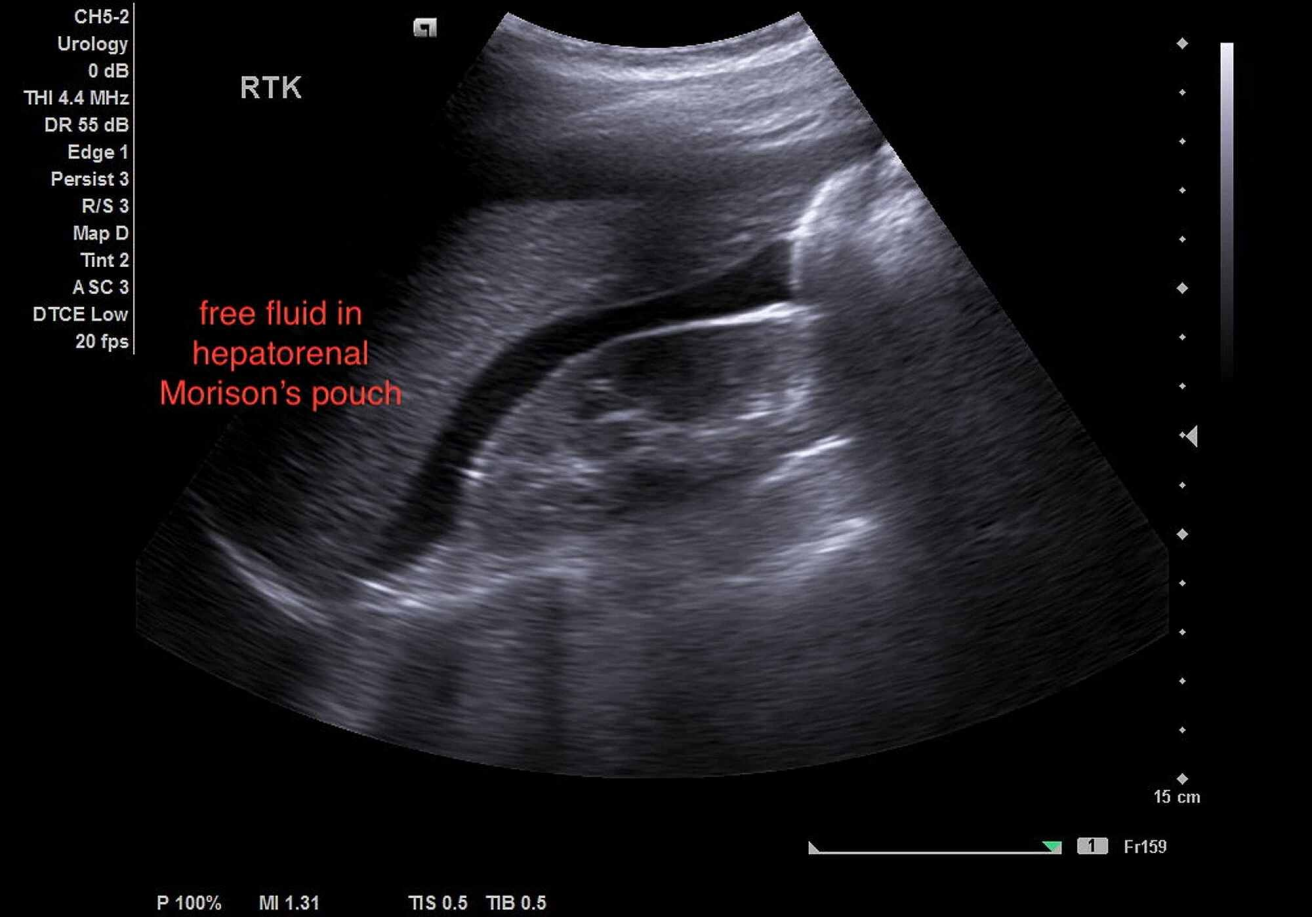
Abdominal US showed free fluids in the peritoneal cavity

A 22-year-old multigravida presented to the emergency department (ED) with dizziness, vomiting, and diffuse abdominal pain at seven weeks of gestation. Her past medical history only included mild blunt abdominal trauma two days ago by her son. There was no history of vaginal bleeding. On the ED initial examination, the patient had stable vital signs and routine blood work. After a few hours, the ED called the surgical team to re-evaluate the patient's sudden hemodynamic instability. The clinical review revealed a confused patient with tachycardia and hypotension (85/40 mmHg). Abdominal examination revealed a distended and tender abdomen. Consequently, we initiated fluid resuscitation with lactated Ringer's solution and ordered adequate cross-matched blood. Abdominal ultrasound (US) revealed marked free fluid in the peritoneal cavity (Figure 1). 

**Figure 2:**
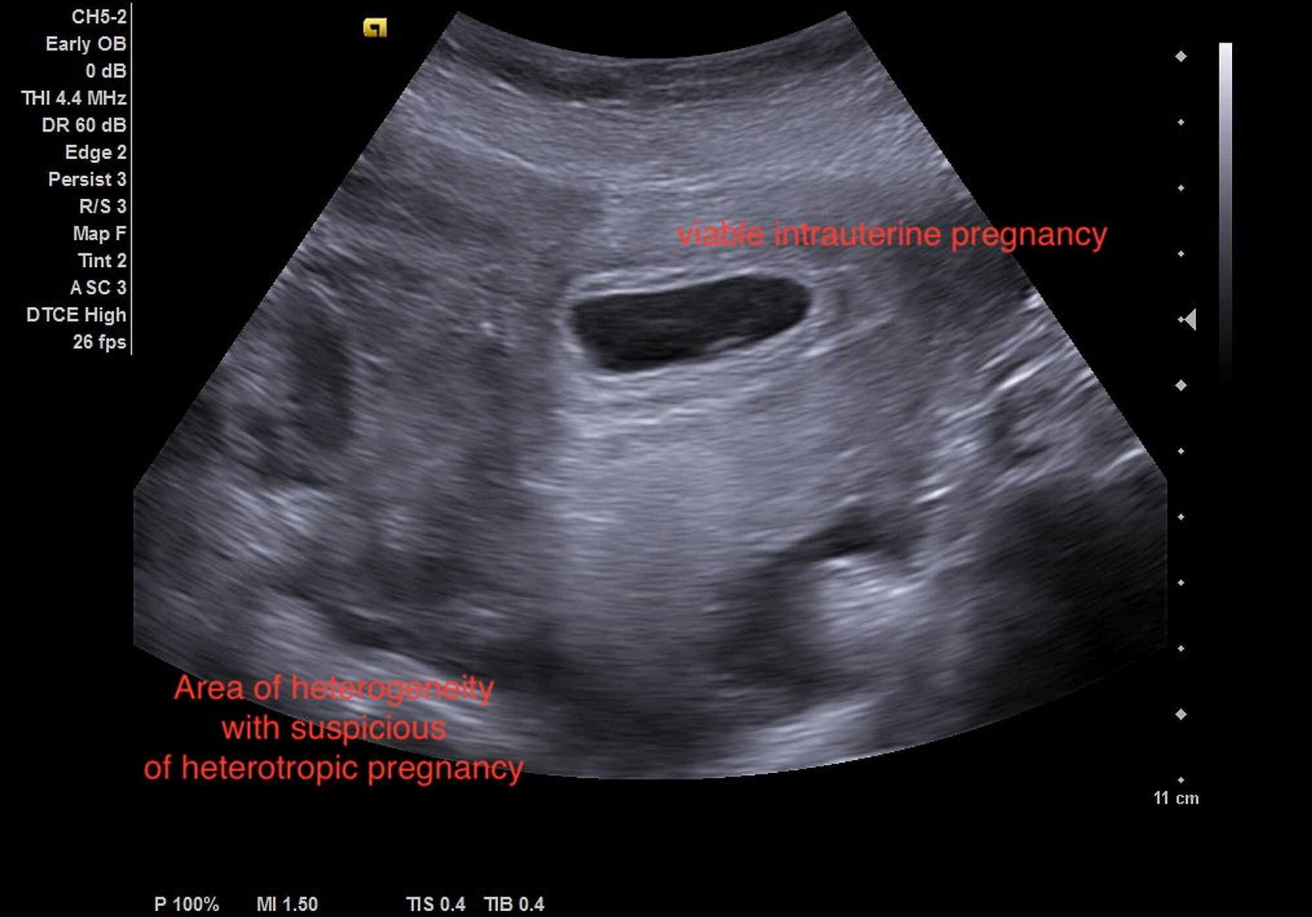
The preoperative US showed a single viable intra-uterine fetus and the heterogeneous area, which raise the suspicious of heterotopic pregnancy

A single viable intrauterine fetus associated with an area of heterogonous echo pattern mass related to the right adnexa surrounded by fluid collection was also evident by sonographic examination, suggesting the possibility of heterotopic pregnancy (Figure 2).

**Figure 3:**
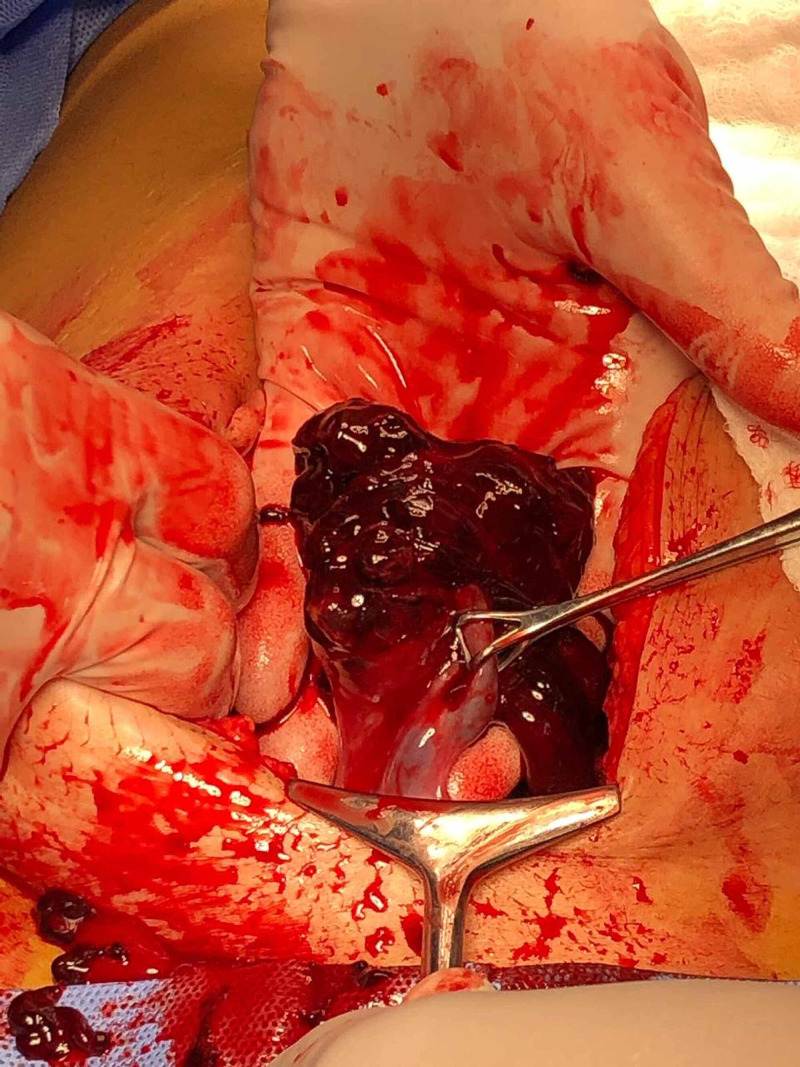
Surgical exploration with evident right-sided ruptured ectopic pregnancy

We opted for immediate diagnostic exploration instead of transferring her to a maternity hospital, given the patient's hemodynamic instability. Intraoperative findings were indicative of marked hemoperitoneum and right-sided ruptured ectopic pregnancy (Figure 3).

**Figure 4:**
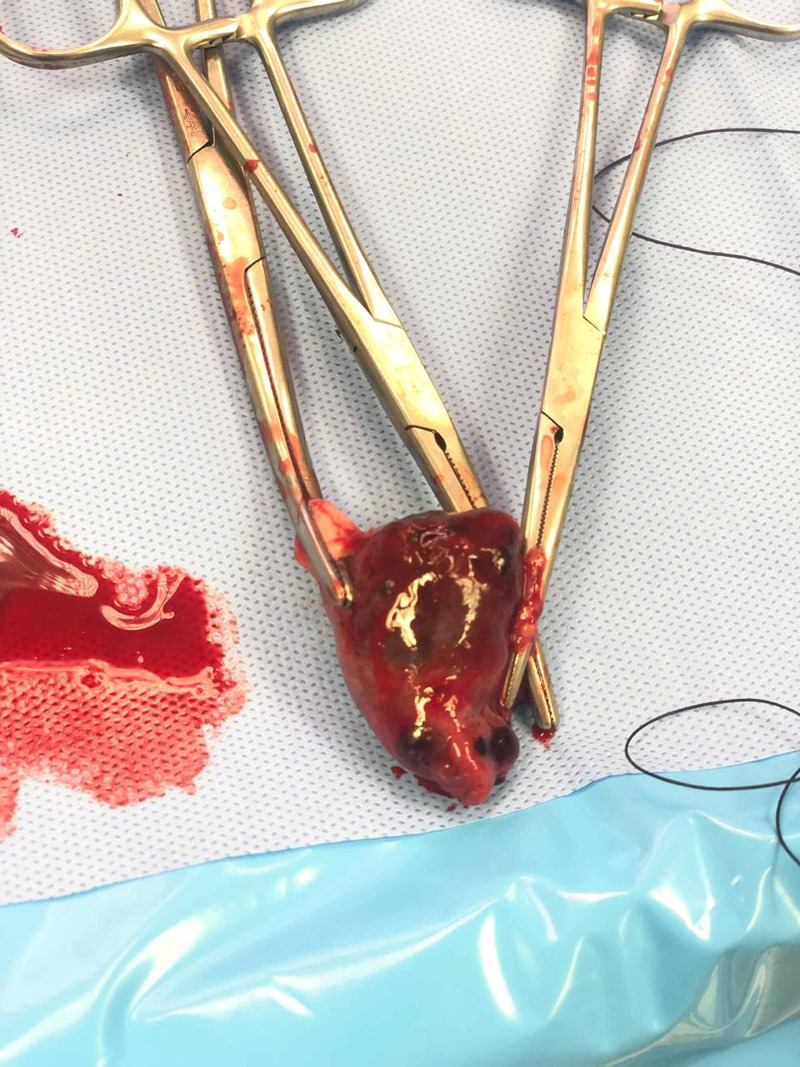
Right salpingectomy specimen along with the ectopic pregnancy tissue

We decided to perform the right salpingectomy with adequate peritoneal lavage (Figure 4).

**Figure 5:**
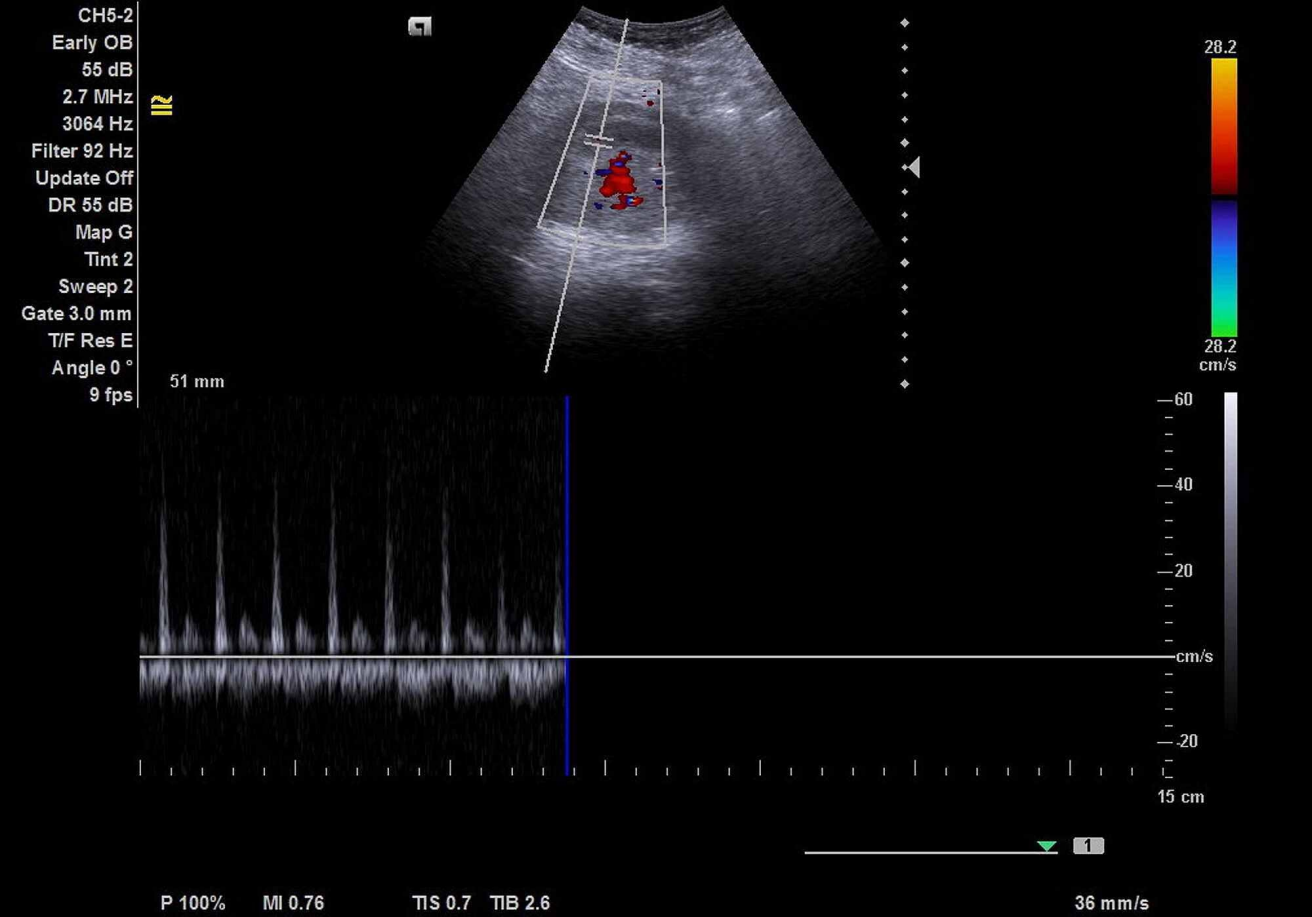
The abdominal US showed fetal heart rate in viable intra-uterine fetus during the early postoperative period.

The patient had an uneventful recovery and was discharged five days after surgery without any complications (Figure 5).

Postoperative histological examination of the specimen confirmed the diagnosis of ruptured ectopic pregnancy. Finally, the patient was followed up in the surgical outpatient clinic and attended regular obstetric follow-up sessions until safe delivery.

## Discussion

Ruptured heterotopic pregnancy is a rare obstetric emergency that may be difficult for inexperienced physicians to diagnose and therefore requires a high index of suspicion. Up to 70% of heterotopic pregnancies can be detected between five to eight weeks of gestation [9]. With the growing popularity of assisted reproductive technology and ovarian hyperstimulation, more patients with heterotopic pregnancy present to emergency care settings, most probably due to the increased hydrostatic pressure during embryo transfer [1,10-12]. 

Our patient had none of the predisposing factors for heterotopic pregnancy but reported a conflicting history of blunt abdominal trauma, which misled the attending ED physician who treated the patient's complaints as expected early pregnancy symptoms. The right adnexal heterogenous mass alongside peritoneal free fluid and intact solid organs excluded that conflicting history on abdominal US examination. In such cases, high-resolution transvaginal US combined with color Doppler can be used as an excellent diagnostic tool because it produces a better visualization of the trophoblastic tissue with typically high flow and low resistive indices. Although this imaging modality is recommended for high-risk patients, particularly those in early pregnancy, its specificity ranges from 26.3% to 92.4% [9,13,14]. 

Many conditions can mimic heterotopic pregnancy - especially when examined by US - such as intrauterine pregnancy associated with corpus luteum cysts, hemorrhagic cysts, and bicornuate uterus gestation. Other acute diseases, like acute appendicitis and cholecystitis, in pregnancy, can also delay the diagnosis [15].

The timing of the onset and presentation of symptoms largely determine the modality of treatment for heterotopic pregnancy. While pharmacological interventions (e.g., potassium chloride or hyperosmolar glucose) and ultrasound-guided interventions can be undertaken in stable patients, surgical intervention represents the most adopted and effective therapy for heterotopic pregnancy [16,17]. The surgical options in emergency settings include salpingectomy, salpingotomy, oophorectomy, or (on rare occasions) hysterectomy [18].

Furthermore, the surgical team can employ either an open or laparoscopic approach depending on the patient's clinical status, surgeon's experience, and surgical instrument availability. Both of these approaches can lead to favourable postoperative outcomes with minimal morbidity. In the presented case, we urge open exploration due to the patient's hemodynamic instability aiming to save the mother's life and viable fetus. Right salpingectomy with minimal manipulation of the gravid uterus was adequate for bleeding control. The cooperation between the surgeons and obstetricians was present from the patient diagnosis and during the patient's follow-up in the postoperative period, which undoubtedly led to a better outcome.

## Conclusions

The current report is a unique example describing how much patient history can be conflicting for the examining physicians and can delay the diagnosis. Also, while heterotopic pregnancy is rare, acute care surgeons must consider the diagnosis during pregnancy, significantly if associated with acute abdomen.
